# Transcriptional Reshaping of Bacteriocytes in the Aphid–*Serratia* Symbiosis

**DOI:** 10.3390/insects17080815

**Published:** 2026-08-05

**Authors:** Yaonian Chen, Xuefeng Jiang, Dening Wang, Qing Dong, Xiaona Zhang, Yifeng Wang, Wenbing Ye

**Affiliations:** 1 College of Agriculture and Biological Engineering, Longnan Normal University, Longnan 742500, China; chenyn0935@163.com (Y.C.); 18298357463@163.com (X.J.); wangdn6085@163.com (D.W.); dqinglwyx@163.com (Q.D.); zxn0706@163.com (X.Z.); wangyifeng0305@163.com (Y.W.); 2 College of Eco-Environmental Engineering, Qinghai University, Xining 810016, China

**Keywords:** aphid, *Serratia symbiotica*, bacteriocyte, transcriptome

## Abstract

The facultative endosymbiont *Serratia symbiotica* significantly influences the ecological fitness of its aphid host. However, the molecular mechanisms by which *Serratia* affects the host’s symbiotic organ, the bacteriocyte, remain poorly understood. Here, we conducted a comparative transcriptomic analysis of bacteriocytes from *Serratia*− and *Serratia*+ pea aphid (*Acyrthosiphon pisum*) strains. Our analysis revealed that *Serratia* colonization extensively modulates gene expression within bacteriocytes. Key metabolic pathways were significantly altered: genes involved in ribosomal biogenesis and oxidative phosphorylation were upregulated, while those in fatty acid biosynthesis were downregulated. Furthermore, we observed a complex reshaping of the immune profile, characterized by a broad downregulation of immune recognition and signaling components alongside an upregulation of specific effector genes and antioxidant enzymes. These findings suggest that *Serratia* induces a state of enhanced anabolic capacity and energy production in bacteriocytes, coupled with strategic reallocation of resources and a finely tuned immune response that balances symbiont tolerance with control. Our RT-qPCR validation confirmed the RNA-seq results, further supporting these results. This study provides the first bacteriocyte-specific transcriptomic resource for the aphid–*Serratia* interaction system, offering novel insights into the molecular integration of a facultative symbiont into host physiology.

## Simple Summary

This study explored how a facultative symbiont, *Serratia symbiotica*, living inside pea aphids, changes the transcriptional level of genes in aphid bacteriocytes. We aimed to understand the molecular details of this symbiotic relationship because it affects the aphid’s environmental adaptations. By comparing transcriptome of bacteriocytes between aphids with and without *Serratia*, we discovered that the symbiont dramatically reshapes the host bacteriocytes’ activity. It upregulates expression of genes related to protein translation and energy production, while downregulating those for fatty acid biosynthesis. The insect’s immune response in these bacteriocytes is also finely adjusted: general immune pathway genes are downregulated, but specific immune effectors are enhanced. These changes suggest the aphid actively manages the symbiont *Serratia*, reallocating its resources to benefit from the partnership while keeping it under control. Our findings provide fundamental insights into how insects and symbionts form stable, beneficial alliances, which is crucial for understanding natural ecosystems and potentially managing agricultural pests.

## 1. Introduction

Throughout long-term co-evolution, insects and their symbionts have established stable, mutualistic relationships, forming a canonical holobiont system in nature [1]. In these relationships, insects provide a suitable habitat for the bacteria, while the symbionts enhance the host’s ecological adaptability across multiple levels [2]. Based on their degree of integration and shared evolutionary history, insect symbionts are classified as either obligate (primary) or facultative (secondary). Obligate symbionts have undergone ancient, specialized co-evolution with their hosts. They reside exclusively within specialized host cells called bacteriocytes, provide essential nutrients to the host, and are strictly vertically transmitted [3]. In contrast, facultative symbionts maintain a more dynamic and evolutionarily recent relationship with their hosts, often lacking strict co-speciation [1]. These symbionts can exhibit traits characteristic of environmental microbes, including the ability to invade various host tissues. From the host’s perspective, these associations are typically conditional; while often beneficial, they can also impose physiological costs [4]. Facultative symbionts can be vertically transmitted via host reproduction and are also capable of horizontal transfer within and between host species [5].

Aphids are globally significant pests whose ecological success is profoundly driven by a rich community of symbionts. These symbionts influence diverse aspects of host biology, including host metabolism, life-history traits, stress tolerance, environmental adaptation, and trophic interactions [6,7,8,9,10]. Most aphids harbor a single obligate symbiont, *Buchnera aphidicola*, which resides exclusively within bacteriocytes. *Buchnera* shares the oldest and most stable symbiotic relationship with its aphid host, and is strictly vertically transmitted [1]. Despite extensive genome reduction due to prolonged co-evolution, *Buchnera* has retained pathways for synthesizing essential amino acids and vitamins, thereby supplying these critical nutrients to aphids that feed on phloem sap—a diet inherently poor in these compounds [10]. In contrast to the obligate *Buchnera*, aphids can also be infected by various facultative symbionts [11]. This diversity underlies a wide range of functions, enabling facultative symbionts to modulate host traits such as metabolism [12], phenotype [13], immune response [14], stress resistance [7], and host plant suitability [9], which ultimately shapes the ecological fitness of their aphid hosts.

*Serratia symbiotica* is a widely distributed and highly diversified facultative symbiont found in numerous aphid populations [15,16,17]. In the pea aphid *Acyrthosiphon pisum*, *Serratia* typically resides within the sheath cells surrounding the bacteriocytes. At this evolutionary stage, it lacks the capacity for independent extracellular life or horizontal transmission, indicating a high level of host adaptation [18]. Although it cannot replace *Buchnera* in providing essential nutrients to the host, a previous study has shown that *Serratia* can invade the bacteriocytes from the sheath cells under specific conditions [19]. This suggests a close and potentially evolving interaction with the primary symbiont, possibly marking a transition toward a co-obligate symbiosis. However, the precise molecular mechanisms underlying the interaction between *Serratia* and the host bacteriocyte remain largely unknown.

Therefore, to elucidate how *Serratia* colonization reshaping bacteriocytes function at the transcriptional level, we conducted a bacteriocyte-specific comparative transcriptomic analysis between *Serratia*-free (*Serratia*−) and *Serratia*-harboring (*Serratia*+) pea aphid strains. Our analysis revealed extensive transcriptional change in host bacteriocytes. GO and KEGG pathway enrichment analyses highlighted a significant upregulation of genes associated with the ribosome and oxidative phosphorylation pathways, coupled with a marked downregulation of genes in the fatty acid biosynthesis pathway. Intriguingly, we also observed substantial alterations in the expression profiles of immune-related genes. This study provides a foundational transcriptomic framework for understanding the interaction between *Serratia* and the aphid bacteriocyte, offering novel insights into the symbiosis systems.

## 2. Materials and Methods

### 2.1. Insect Rearing

A pea aphid colony, naturally only harboring the obligate symbiont *Buchnera* and the facultative symbiont *Serratia*, was collected in 2020 from a *Medicago sativa* field in Yunnan, China. The presence of the facultative symbiont in this colony was screened using diagnostic PCR by specific primers (Table S1). The aphid colony was maintained on broad bean *Vicia faba* plants under controlled conditions at 22 °C, 70% relative humidity, and a 16:8 h (light:dark) photoperiod.

### 2.2. Establishment of Serratia− and Serratia+ Aphid Strains

The original laboratory aphids were microinjected with 5 μg ampicillin using a Nanoliter 2010 injector system (World Precision Instruments Inc., Sarasota, FL, USA). The injected aphids were designated as G0 and were reared on *V. faba* seedlings separately. Eight G1 nymphs from G0 adults were randomly selected and were also maintained on *V. faba* seedlings separately. This process was repeated for one G2 nymph from each G1 adult, and the same process was repeated for the subsequent generations of aphids. PCR was conducted for the screening of *Serratia* in each generation of aphids. The specific primers were listed in Table S1. We established a stable *Serratia*− aphid strain in the 6th generation. This method referred to previous study [20].

To obtain the *Serratia*+ aphid strain, we microinjected hemolymph from original laboratory aphids into *Serratia*− aphids. In detail, aphid legs were dissected, and the flowing hemolymph was pipetted. Hemolymph from a total of fifty original laboratory aphids were harvested and was microinjected into fifty 2nd-instar *Serratia*− nymphs (50 nL per nymph). Injected aphids were maintained on *V. faba* seedlings separately. The G1 nymphs were subjected to PCR, and the aphids harboring *Serratia* were reared on fresh *V. faba* seedlings to re-establish the *Serratia*+ aphid strain. Both aphid strains were maintained on broad bean *V. faba* plants under controlled conditions at 22 °C, 70% relative humidity, and a 16:8 h (light:dark) photoperiod. Both aphid strains were maintained for more than 10 generations before conducting further experiments.

### 2.3. Whole-Mount Fluorescence In Situ Hybridization

Localization of symbionts was performed using whole-mount fluorescence in situ hybridization (FISH). The 3rd-instar nymphs were fixed in Carnoy’s fixative (ethanol:chloroform:acetic acid, 6:3:1) at room temperature for 24 h. Fixed samples were washed in 100% ethanol and bleached in 6% H_2_O_2_ (in ethanol) at room temperature for 10 days. After bleaching, samples were washed in 70% ethanol, and then legs were removed. Samples were rehydrated in 0.3% PBST (PBS with 0.3% Tween 20) and incubated in pre-hybridization buffer (20 mM Tris-HCl pH 8.0, 0.9 M NaCl, 0.01% SDS, 30% formamide) at 46 °C for 30 min. Hybridization was performed overnight at 46 °C in the same buffer containing 100 nM of each fluorescently labeled, symbiont-specific DNA probe (targeting *Serratia* or *Buchnera* 16S rRNA). Probes were listed in Table S1. After hybridization, samples were washed in 0.3% PBST, counterstained with DAPI (1:2000 dilution in PBST), and mounted on slides for observation. Specimens were imaged using a laser scanning confocal microscope and analyzed with ZEN software v2.3 (Carl Zeiss Microscopy GmbH, Jena, Germany).

### 2.4. PCR and Absolute Quantitative PCR

For DNA extraction, individual aphids were surface-sterilized in 75% ethanol and washed in PBS. Total genomic DNA was extracted using the TIANamp Genomic DNA Kit (Tiangen, Beijing, China). Diagnostic PCR with *Serratia*-specific primers was used for screening of the symbiont’s presence. The thermal profile was: 95 °C for 2 min; 35 cycles of 95 °C for 30 s, 55 °C for 30 s, and 72 °C for 1 min; and a final extension at 72 °C for 5 min.

Absolute qPCR was used to quantify symbiont titers [20]. The single-copy dnaK genes from *Buchnera* and *Serratia*, and the aphid single-copy nuclear gene elongation factor 1-α (EF1α), were amplified from whole-aphid DNA. Standard curves were generated using ten-fold serial dilutions (102 to 108 copies) of plasmid DNA containing each target gene. Reactions were performed in a CFX Connect Real-Time PCR Detection System (BioRad, Hercules, CA, USA) using 2×SYBR Premix Ex TaqII (Takara, Kusatsu, Japan) with specific primers. The thermal profile was: 95 °C for 15 min; 40 cycles of 95 °C for 15 s and 60 °C for 1 min; followed by a melt curve analysis.

### 2.5. Bacteriocyte Collection and RNA Extraction

Bacteriocytes were manually dissected from adult aphids of both strains in ice-cold PBS. For each biological replicate, bacteriocytes from 40 aphids were pooled. Three independent biological replicates were collected per aphid strain. Total RNA was extracted from the pooled tissues using TRIzol Reagent (Invitrogen, Carlsbad, CA, USA) according to the manufacturer’s protocol. RNA concentration and integrity were assessed, and samples were stored at −80 °C.

### 2.6. cDNA Library Construction and RNA-Seq Sequencing

RNA-seq libraries were constructed using a stranded, poly(A)-enrichment protocol. Briefly, mRNA was enriched from total RNA using oligo(dT) magnetic beads, fragmented, and reverse-transcribed using random hexamers. Double-stranded cDNA was synthesized, end-repaired, adenylated, and ligated to sequencing adapters. The ligated products were PCR-amplified, and the final libraries were quality-controlled. Qualified libraries were used to generate DNA nanoballs, which were loaded onto patterned nanoarrays and sequenced on the DNBSEQ-T7 platform (MGI Tech Co., Ltd., Shenzhen, China), generating 150 bp paired-end reads.

### 2.7. RNA-Seq Analysis

Raw sequencing reads were processed to obtain clean data by removing adapter sequences, reads containing poly-N, and low-quality reads using in-house Perl scripts. Quality metrics, including Q20, Q30 scores, and GC content, were calculated. Clean reads were then mapped to the pea aphid reference genome (assembly: pea_aphid_22Mar2018_4r6ur_v2) using HISAT2. Gene expression levels were quantified as Fragments Per Kilobase of transcript per Million mapped reads (FPKM). Principal component analysis (PCA) and sample correlation heatmaps were generated based on normalized expression values. The raw data were uploaded to NCBI with the accession number PRJNA1419713.

Differential expression analysis was performed using DESeq2. Genes with an absolute fold change ≥ 2 (|log_2_FC| ≥ 1) and a false discovery rate (FDR) < 0.05 were considered significantly differentially expressed. Gene function was annotated using the NCBI non-redundant (Nr), Swiss-Prot, KEGG, Gene Ontology (GO), and Clusters of Orthologous Groups (COG) databases. Statistical enrichment of differentially expressed genes (DEGs) in KEGG pathways and GO terms was tested.

### 2.8. RT-qPCR Validation

To validate the RNA-seq data, we selected ten DEGs representing the main functional groups. Total RNA of bacteriocytes was extracted from the four biological replicates. cDNA was synthesized using PrimeScript RT Reagent Kit (Takara). qRT-PCR was performed with CFX Connect system. The aphid *EF1α* and *PRS20* gene was used as the internal control. Relative expression was calculated by the 2^−ΔΔCt^ method. Student’s *t*-test was applied to determine significance (*p* < 0.05). Primer sequences are provided in Table S1.

### 2.9. Statistical Analysis

All the statistical analyses were performed using GraphPad Prism v8.0 (GraphPad Software) and SPSS 26 (IBM). The normality of the distribution of the data was tested using the Shapiro–Wilk test. We used Student’s *t* tests to determine the statistical significance of symbiont titer differences between *Serratia*− and *Serratia*+ aphid strains.

## 3. Results

### 3.1. Establishment of Serratia− and Serratia+ Aphid Strains

**Figure 1:**
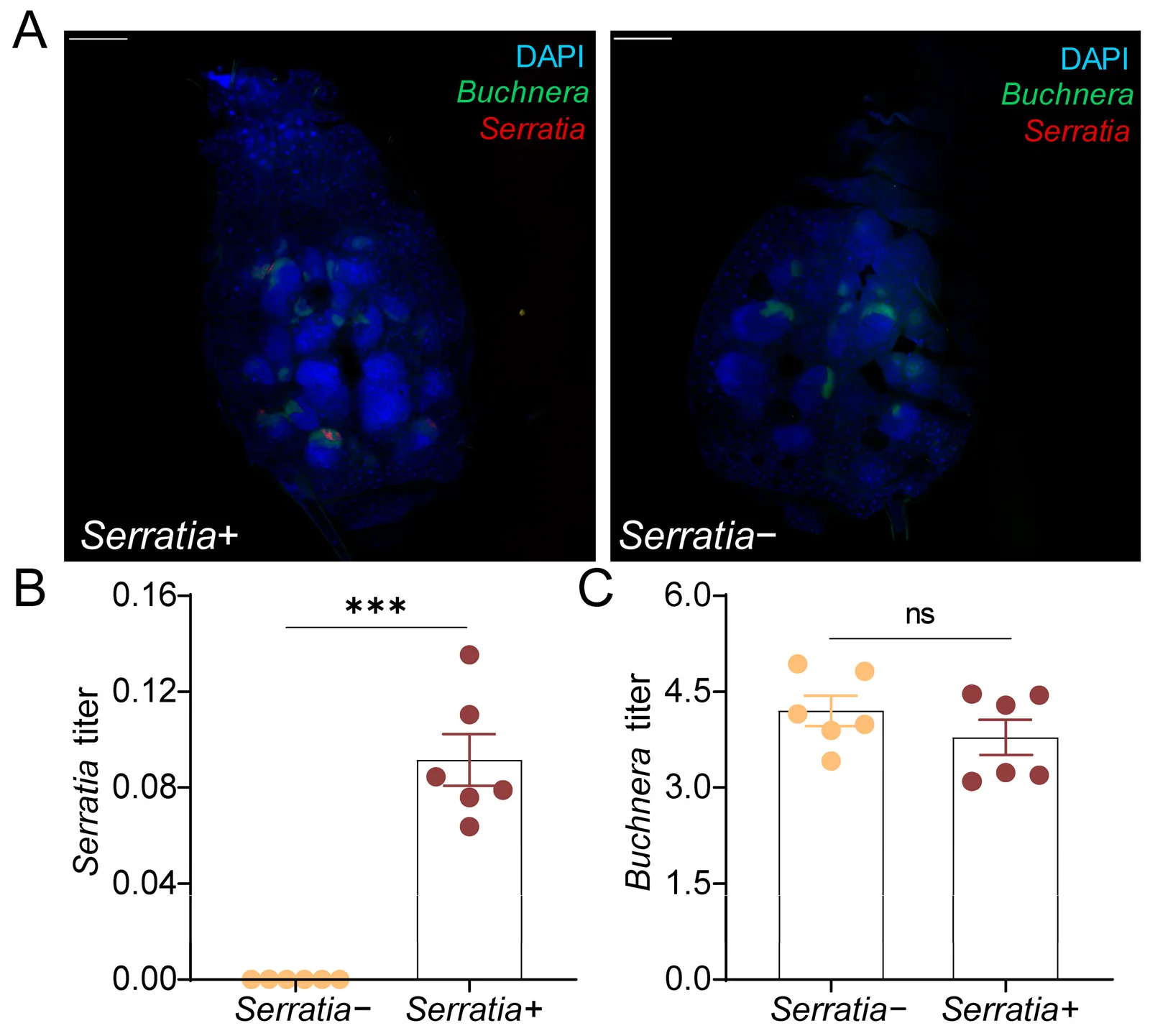
Establishment of *Serratia*− and *Serratia*+ aphid strains. (**A**) Whole-mount FISH analysis of each aphid strain to detect *Buchnera* and *Serratia*. *Buchnera*, green; *Serratia*, red; aphid DNA (DAPI), blue. Scale bar = 0.2 mm. (**B**) *Serratia* titer in *Serratia*− or *Serratia*+ aphid strain. (**C**) *Buchnera* titer in *Serratia*− or *Serratia*+ aphid strain. For (**B**,**C**), data are means ± SEM from six biological replicates represented by the scatter points. Statistical significances were determined by Student’s *t*-test (*** *p* < 0.001; ns, not significant).

We firstly screened the presence of facultative symbionts in our pea aphid colony using diagnositic PCR. Results showed that the aphid colony only harbors the obligate symbiont *Buchnera* and the facultative symbiont *Serratia* (Figure S1). To generate a *Serratia*-free aphid strain, ampicillin was microinjected into original laboratory aphids (naturally harboring *Serratia*). Individual aphids (G0) were then isolated and allowed to reproduce. The offspring were screened via PCR for the absence of *Serratia* infection to establish a stable *Serratia*− aphid strain. To re-establish the *Serratia*+ aphid strain, hemolymph from original laboratory aphids was microinjected into the *Serratia*− aphids. Their offspring were similarly screened by PCR to obtain a stable *Serratia*+ aphid strain. Whole-mount FISH confirmed that *Serratia* specifically resided in the sheath cells surrounding the bacteriocytes in the *Serratia*+ aphid and was absent in the *Serratia*− aphid (Figure 1A). Furthermore, absolute quantitative PCR verified the complete absence of *Serratia* in the *Serratia*− aphid strain (Figure 1B), while the titer of the primary endosymbiont *Buchnera* did not differ significantly between *Serratia*− and *Serratia*+ aphid strains (Figure 1C).

### 3.2. Overview of the RNA-Seq Data

To analyze the transcriptional changes in bacteriocytes between the *Serratia*− and *Serratia*+ aphid strains, we performed RNA-seq on bacteriocytes from both strains. Following quality control, 76.07% to 91.51% of the clean reads were uniquely mapped to the pea aphid reference genome. The overall sequencing data had an average GC content of 40.45% and an average Q30 score of 97.10% (Table S2). A total of 16,654 genes were identified across all six samples. The expression level of each gene was calculated and normalized using the FPKM method.

### 3.3. Differential Expression Analysis

**Figure 2:**
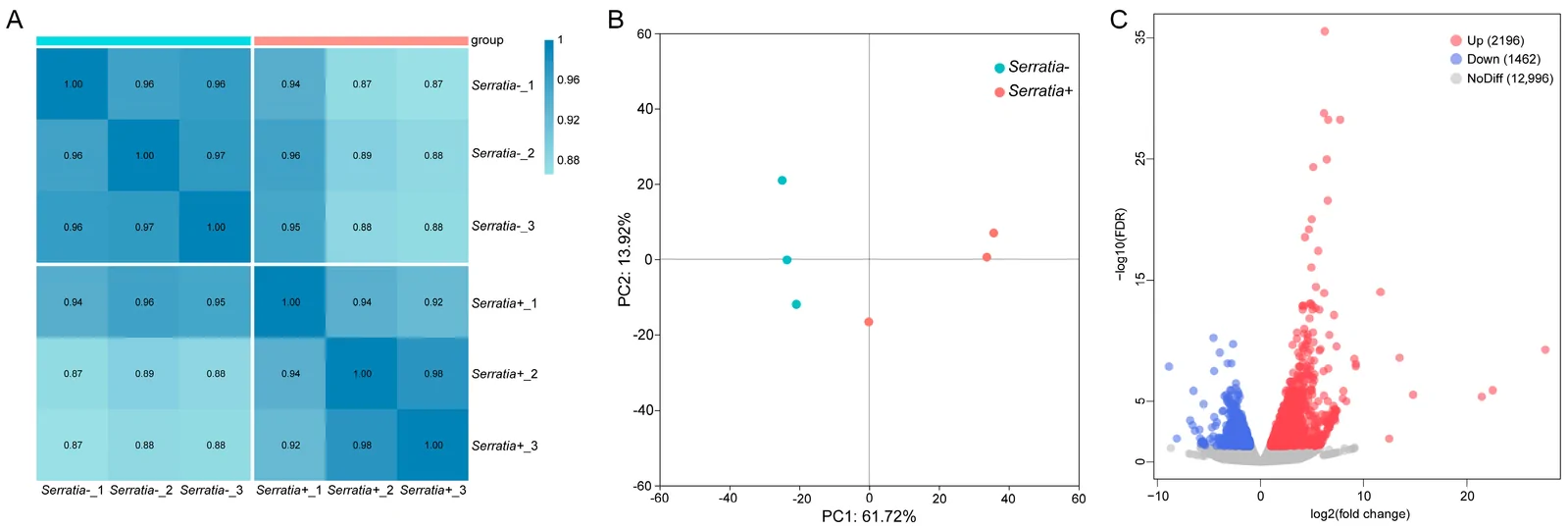
Differential expression analysis of *Serratia*− and *Serratia*+ aphid bacteriocytes. (**A**) Correlation heatmap of *Serratia*− and *Serratia*+ aphid bacteriocyte samples. The cyan and red annotation bars indicate the *Serratia*− and *Serratia*+ groups, respectively, and the blue color scale represents the pairwise correlation coefficients between samples. (**B**) PCA of *Serratia*− and *Serratia*+ aphid bacteriocyte samples. (**C**) Volcano plot showing the DEG *Serratia*− and *Serratia*+ aphid bacteriocyte samples (FDR < 0.05 and fold change ≥ 2, Benjamini–Hochberg FDR method).

PCA and the correlation heatmap demonstrated high intra-group similarity and clear inter-group separation among six samples, indicating reliable replicates and substantial transcriptional differences (Figure 2A,B). We identified 3658 DEGs (|log2(Fold change)| > 1; FDR < 0.05), with 2196 upregulated and 1462 downregulated in *Serratia*+ bacteriocytes compared to *Serratia*− ones (Figure 2C).

**Figure 3:**
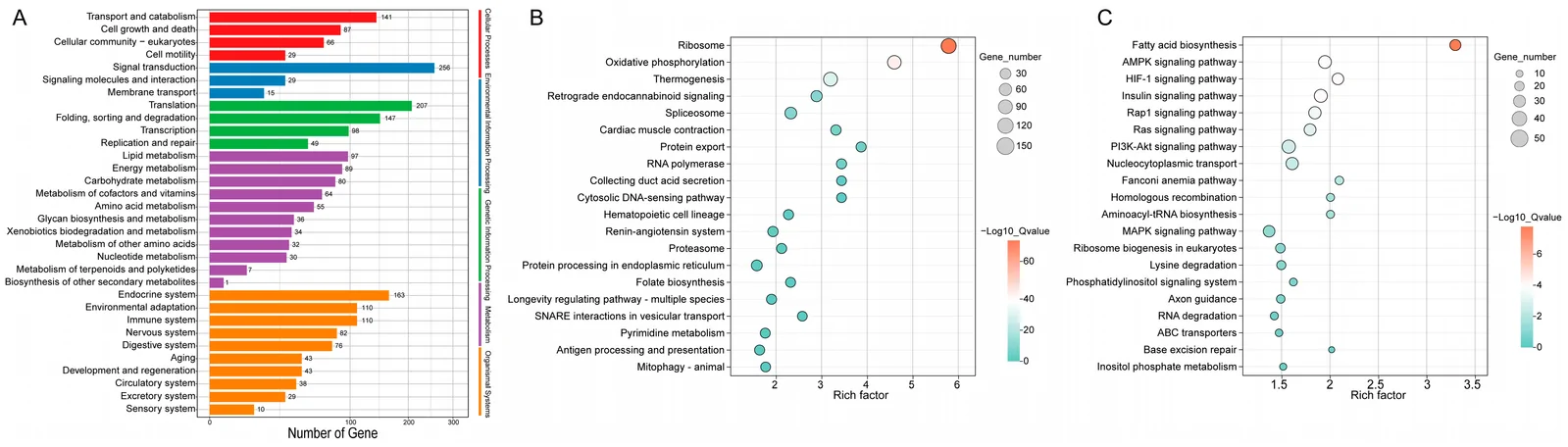
KEGG pathway analysis of DEGs. (**A**) KEGG pathway classification for all DEGs. (**B**) KEGG significant enrichment analysis for the upregulated genes. (**C**) KEGG significant enrichment analysis for the downregulated genes.

To investigate the potential biological functions of the DEGs, GO and KEGG enrichment analyses were performed. For the GO classification (Q Value ≤ 0.05), the DEGs were categorized into three ontologies: biological process, cellular component, and molecular function. Within the biological process ontology, the term with the highest number of assigned DEGs was “cellular process” (1142 genes). In the cellular component ontology, it was “cellular anatomical entity” (1652 genes), and in the molecular function ontology, the term “binding” was the most enriched (1154 genes) (Figure S2). The DEGs were also mapped to KEGG pathways. A total of 2353 genes were assigned to 32 pathway terms (Figure 3A). Among these, the terms “signal transduction” and “translation” were the largest groups, containing 256 and 207 genes, respectively. Notably, KEGG pathway enrichment analysis revealed that the “Ribosome” and “Oxidative phosphorylation” pathways were significantly enriched among the upregulated genes (involving 110 and 83 genes, respectively) (Figure 3B). In contrast, the “Fatty acid biosynthesis” pathway was significantly enriched among the downregulated genes (involving 26 genes) (Figure 3C). Collectively, the GO and KEGG analyses provide a systematic overview of the functions associated with the transcriptional changes, highlighting key pathways that are likely to play important roles in the functional modulation of aphid bacteriocytes associated with the presence of *Serratia*.

### 3.4. Upregulation of Gene Expression in the Ribosome and Oxidative Phosphorylation Pathway

**Figure 4:**
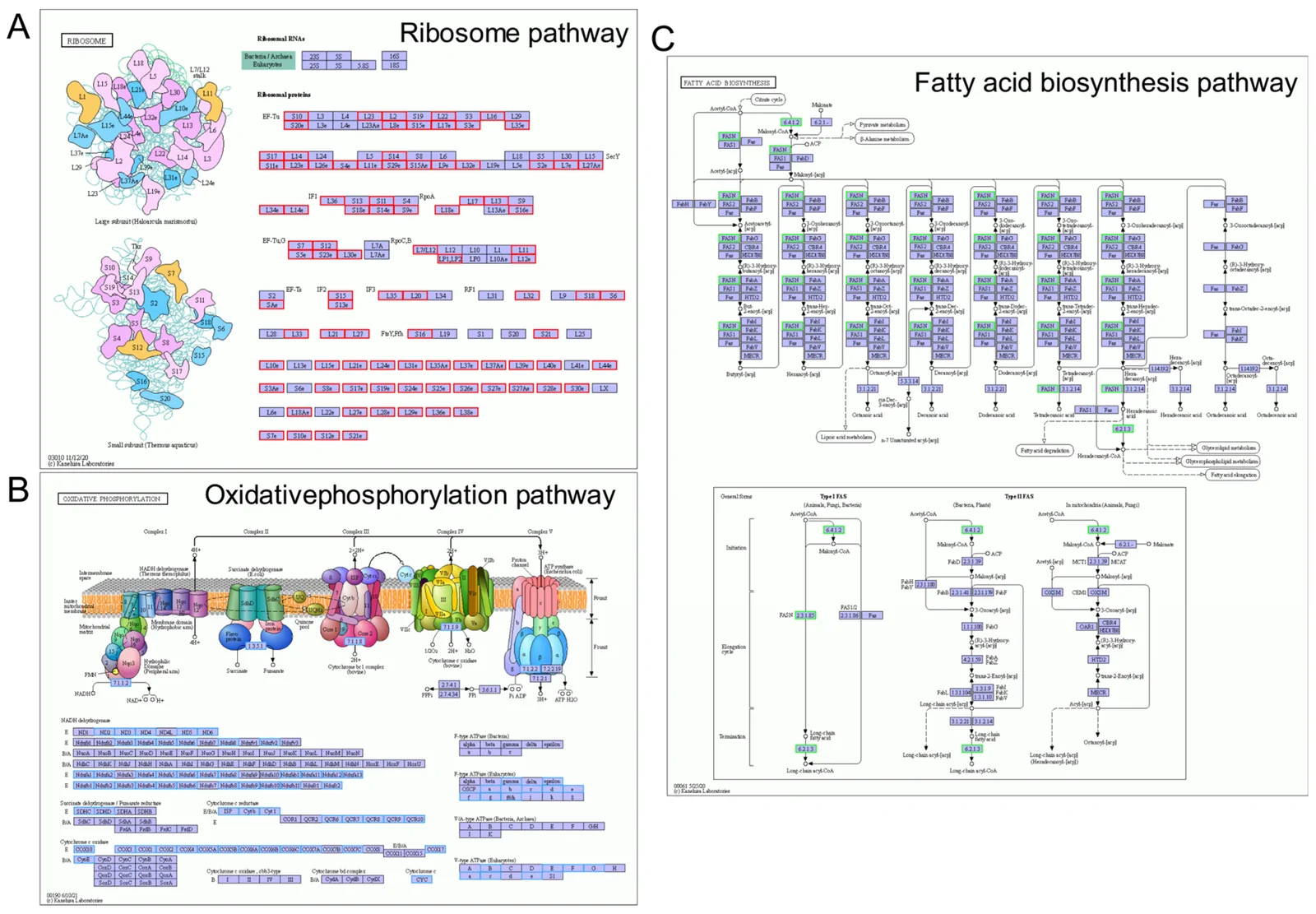
The KEGG pathways showing transcriptional responses to *Serratia* colonization. (**A**) The KEGG pathway of the ribosome pathway responds to *Serratia* colonization. Genes highlighted in red are enriched and upregulated. (**B**) The KEGG pathway of the oxidative phosphorylation pathway responds to *Serratia*. Genes highlighted in blue are enriched and upregulated. (**C**) The KEGG pathway of the fatty acid biosynthesis pathway responds to *Serratia*. Genes highlighted in green are enriched and downregulated.

Numerous genes encoding ribosomal proteins, including components of both the large and small subunits, were significantly upregulated in the bacteriocytes of *Serratia*+ aphids (Figure 4A and Table S3). Meanwhile, a marked upregulation was observed for genes involved in the oxidative phosphorylation pathway in the bacteriocytes of *Serratia*+ aphids (Figure 4B and Table S4). This included genes encoding key enzyme complexes such as NADH dehydrogenase, succinate dehydrogenase, cytochrome c reductase, cytochrome c oxidase, and ATPase. These results suggest that bacteriocytes harboring *Serratia* undergo a metabolic reshaping characterized by enhanced translational capacity and energy production, which may collectively contribute to greater cellular adaptation under stress.

### 3.5. Downregulation of Gene Expression in the Fatty Acid Biosynthesis Pathway

A large number of genes involved in fatty acid biosynthesis were significantly downregulated in the bacteriocytes of *Serratia*+ aphids (Figure 4C), specifically the genes encoding key enzymes such as long-chain fatty acid CoA ligase, acetyl-CoA carboxylase, and fatty acid synthase (Table S5). This downregulation suggests that aphids harboring *Serratia* may reduce or even cease autonomous fatty acid production within their bacteriocytes, potentially shifting to acquire these essential lipids directly from *Serratia*.

### 3.6. Changes in Expression of Immune-Related Genes

A significant number of genes associated with immunity were identified among DEGs in the bacteriocytes from *Serratia*− vs. *Serratia*+ aphid strains. For recognition receptors, GNBP1 and GNBP2 (Gram-negative binding protein) were significantly downregulated in *Serratia*+ aphid bacteriocytes. Within the canonical immune cascade Toll signaling pathway, key components including one spätzle (extracellular cytokine), three transmembrane Toll receptors, and the transactivator dorsal were also downregulated in *Serratia*+ aphid bacteriocytes. Similarly, the JAK tyrosine kinase (Hopscotch) of the JAK/STAT signaling pathway showed reduced expression. Furthermore, nine genes encoding serine proteases (SPs) within the serine protease cascade were downregulated, but two SP genes (LOC100158977 and LOC100164343) were upregulated (Table 1).

Interestingly, in contrast to the broad downregulation of immune signaling components, the expression of several immune effector genes was significantly upregulated. This included all three lysozyme genes and the majority of bacteriocyte-specific cysteine-rich proteins (BCRs), a class of putative antimicrobial peptides. Consistently, the expression of three genes encoding the core antioxidant enzyme Peroxiredoxin was significantly upregulated in *Serratia*+ aphid bacteriocytes (Table 1). These results indicate profound and complex changes in bacteriocyte immunity associated with the presence of *Serratia*.

### 3.7. RT-qPCR Validation for Some Candidates

**Figure 5:**
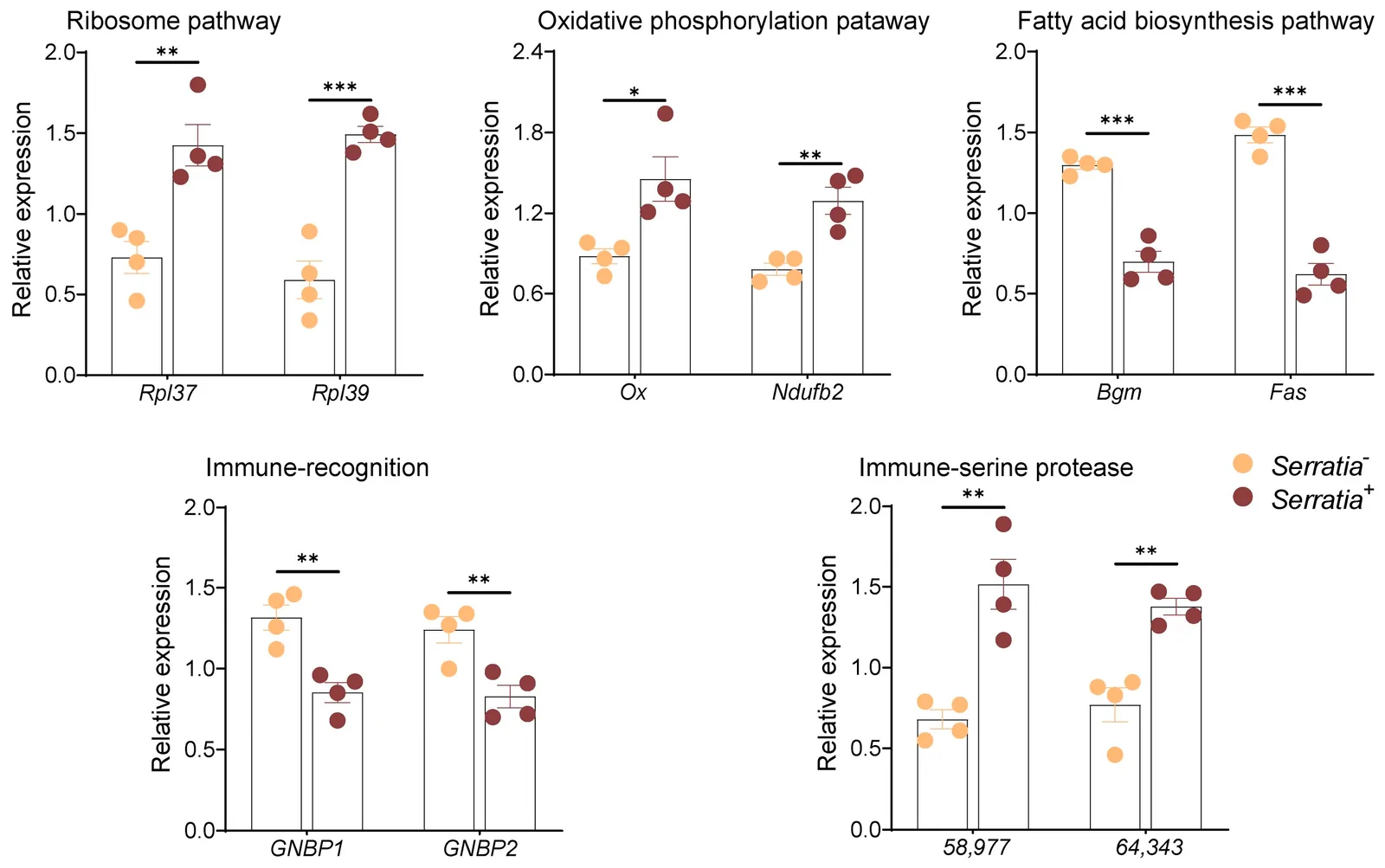
The RT-qPCR expression profiles for selected differentially expressed genes. All RT-qPCR reactions were performed in four biological replicates, and data are shown as means ± SEM. Asterisks indicate significant differences between strains (*p* < 0.05, *; *p* < 0.01, **; *p* < 0.001, ***; Student’s *t*-test).

To corroborate the RNA-seq data, we performed RT-qPCR on a subset of ten DEGs that represent the major functional categories identified in this study. The expression patterns of all tested genes were consistent with the RNA-seq results (Figure 5). For example, the ribosomal protein genes *Rpl37* (LOC100159544) and *Rpl39* (LOC100190998) were significantly upregulated in *Serratia*+ bacteriocytes, as confirmed by RT-qPCR (Figure 5). Similarly, the oxidative phosphorylation gene *OX* (LOC100573220) and *Ndufb2* (LOC100302482) showed significant upregulation, whereas fatty acid biosynthesis genes Bgm (LOC100164756) and Fas (LOC100160920) were significantly downregulated (Figure 5). The immune recognition genes GNBP1 (LOC100164352) and GNBP2 (LOC100165182) exhibited significant downregulation, while two serine protease genes (LOC100158977 and LOC100164343) showed significant upregulation (Figure 5). These qRT-PCR data quantitatively support the reliability of our transcriptomic analysis.

## 4. Discussion

The facultative endosymbiont *Serratia symbiotica*, with its high prevalence and genetic diversity in aphids, serves as an excellent model for investigating insect–symbiont interactions [15,21]. Our study provides the first transcriptomic exploration into the impact of *Serratia* colonization on the host’s symbiotic organ (bacteriocyte), offering a valuable gene expression resource to further elucidate the molecular basis of this symbiosis.

Previous research has demonstrated that *Serratia* enhances aphid ecological fitness by modulating various physiological processes. These include improving heat tolerance, growth, and fecundity, suppressing host plant defenses, and even regulating wing polyphenism to influence population dispersal [12,13,22,23]. Our findings offer mechanistic insights into how such benefits are energetically sustained. The concurrent upregulation of ribosomal biogenesis and oxidative phosphorylation pathways in *Serratia*+ aphid bacteriocytes indicates a state of heightened anabolic activity and energy production. This enhanced metabolic activity likely meets the increased biosynthetic demands of maintaining the additional symbiont, thereby supporting the enhanced physiological performance of the host.

This symbiotic benefit, however, comes with a metabolic trade-off [4,24]. We observed a significant downregulation of the fatty acid biosynthesis pathway in *Serratia*+ aphid bacteriocytes. This finding appears to contrast with earlier reports of induced fatty acid synthase expression upon *Serratia* infection [12]. We posit that this discrepancy highlights a strategic reallocation of resources [25,26]. In the symbiotic state, the host bacteriocyte may reduce its own energetically costly lipid synthesis, reallocating resources towards translation and energy production. The essential lipid requirements may instead be supplemented through cooperation with *Serratia*, exemplifying a deep metabolic integration.

Furthermore, our analysis reveals a sophisticated reshaping of the bacteriocyte immune profile in response to *Serratia* colonization. The broad downregulation of immune recognition (GNBPs) and signaling (Toll, JAK/STAT) components suggests a suppression of canonical immune responses within the bacteriocyte. This systemic downregulation likely represents a host adaptation to tolerate and maintain the facultative symbiont, preventing energetically costly and potentially damaging immune activation against a beneficial partner [27,28,29]. Crucially, this immunosuppression is not absolute but is fine-tuned. The documented potential of *Serratia* to invade bacteriocytes under certain conditions reveals an inherent instability in the symbiosis [15,19,30], necessitating host mechanisms to prevent over-proliferation or inappropriate localization of the symbiont [31,32]. The selective upregulation of specific effectors—such as bacteriocyte-specific cysteine-rich proteins (BCRs) and lysozymes [33,34,35,36]—implies a retained host capacity to monitor and regulate symbiont titers, potentially preventing uncontrolled invasion of the bacteriocyte. The significant upregulation of peroxiredoxins likely serves as a targeted antioxidant response to manage reactive oxygen species produced by the enhanced oxidative phosphorylation, thereby maintaining cellular homeostasis.

In conclusion, *Serratia* colonization drives a comprehensive reshaping of the bacteriocyte at the transcriptional level. This involves investing in core biosynthetic and energetic systems, outsourcing certain metabolic functions, and precisely modulating the immune responses—from broad tolerance to targeted surveillance and homeostasis maintenance. This study reveals the complex transcriptional change underlying stable symbiosis and provides a framework for understanding how facultative partners are integrated into host physiology.

## Figures and Tables

**Table 1:** Differential expression of the immune-related genes in bacteriocytes between two aphid strains.

Gene ID	*Serratia*−_FPKM	*Serratia+*_FPKM	Log2(FC)	Blast nr
**Recognition**				
LOC100164352	12.34	1.80	−2.24	GNBP1
LOC100165182	12.54	0.92	−3.19	GNBP2
**Toll pathway**				
LOC100163265	21.59	4.31	−1.53	Spaetzle 1-1
LOC100161089	5.16	0.88	−1.96	protein toll
LOC100572161	3.98	0.5	−2.27	toll-like receptor 7
LOC100162436	29.72	4.08	−2.24	dorsal
LOC100158739	13.92	1.83	−2.30	protein toll
**JAK pathway**				
LOC100167312	41.57	10.26	−1.15	tyrosine-protein kinase hopscotch
**Serine protease and inhibitor**				
LOC100161864	31.14	5.89	−1.77	modular serine protease
LOC107882158	13.42	2.60	−1.79	Uncharacterized serine protease
LOC100158742	40.22	7.29	−1.77	Serine protease inhibitor
LOC100163968	723.46	163.77	−1.32	Serine protease inhibitor
LOC100573323	13.73	2.80	−1.51	Uncharacterized serine protease
LOC100160715	27.70	4.90	−1.96	Serine protease Hayan
LOC100160985	134.21	30.54	−1.41	Serine protease inhibitor
LOC100166676	13.90	3.23	−1.33	Serine protease inhibitor
LOC100158977	84.57	115.02	1.18	Uncharacterized serine protease
LOC100163730	2.25	0.43	−1.80	Uncharacterized serine protease
LOC100164343	0.99	3.48	2.86	Serine protease masquerade
**Effectors**				
LOC100160909	33,836.84	70,088.78	2.05	Lysozyme
LOC100168424	16.62	33.61	2.06	Lysozyme
LOC100167742	142.41	387.38	2.56	Lysozyme
novel.5244	3245.43	4808.91	1.44	BCR1
novel.5251	6035.59	8826.79	1.46	BCR2
LOC103310324	6894.90	14,782.09	2.25	BCR3
LOC100302494	420.66	740.26	1.75	BCR5
LOC100570675	957.15	1593.88	1.47	BCR6
LOC100574884	3622.50	8057.51	2.32	BCR8
**ROS**				
LOC100168384	494.38	601.05	1.34	Peroxiredoxin 5
LOC100161272	1352.57	1832.01	1.45	Peroxiredoxin 1
LOC100162824	165.55	194.83	1.22	Peroxiredoxin

## Data Availability

All raw data generated by RNA-seq were uploaded to NCBI with the accession number PRJNA1419713.

## Supplementary Materials

The following supporting information can be downloaded at https://www.mdpi.com/article/10.3390/insects17080815/s1: Figures S1 and S2; Tables S1–S5; Figure S1. The screening of facultative symbionts in our pea aphid colony via PCR; Figure S2. The GO classification showing transcriptional responses to *Serratia* colonization (Q Value ≤ 0.05); Table S1. Primers used in this study; Table S2. Data output quality and mapping rates for the examined samples; Table S3. The upregulated genes encoding ribosomal proteins in the bacteriocytes of *Serratia*+ aphids; Table S4. The upregulated genes in oxidative phosphorylation pathway in the bacteriocytes of *Serratia*+ aphids; Table S5. The downregulated genes involved in fatty acid biosynthesis in the bacteriocytes of *Serratia*+ aphids.

## References

[1] Dale C., Moran N.A. (2006). Molecular interactions between bacterial symbionts and their hosts. Cell.

[2] Douglas A.E. (2015). Multiorganismal insects: Diversity and function of resident microorganisms. Annu. Rev. Entomol..

[3] Koga R., Meng X.-Y., Tsuchida T., Fukatsu T. (2012). Cellular mechanism for selective vertical transmission of an obligate insect symbiont at the bacteriocyte–embryo interface. Proc. Natl. Acad. Sci. USA.

[4] Zytynska S.E., Tighiouart K., Frago E. (2021). Benefits and costs of hosting facultative symbionts in plant-sucking insects: A meta-analysis. Mol. Ecol..

[5] Ahmed M.Z., Li S.J., Xue X., Yin X.J., Ren S.X., Jiggins F.M., Greeff J.M., Qiu B.L. (2015). The intracellular bacterium *Wolbachia* uses parasitoid wasps as phoretic vectors for efficient horizontal transmission. PLoS Pathog..

[6] Degnan P.H., Yu Y., Sisneros N., Wing R.A., Moran N.A. (2009). *Hamiltonella defensa*, genome evolution of protective bacterial endosymbiont from pathogenic ancestors. Proc. Natl. Acad. Sci. USA.

[7] Heyworth E.R., Ferrari J. (2015). A facultative endosymbiont in aphids can provide diverse ecological benefits. J. Evol. Biol..

[8] Oliver K.M., Degnan P.H., Hunter M.S., Moran N.A. (2009). Bacteriophages encode factors required for protection in a symbiotic mutualism. Science.

[9] Li Q., Fan J., Sun J.X., Zhang Y., Hou M.L., Chen J.L. (2019). Anti-plant defense response strategies mediated by the secondary symbiont *Hamiltonella defensa* in the wheat aphid *Sitobion miscanthi*. Front. Microbiol..

[10] Douglas A.E. (1998). Nutritional interactions in insect-microbial symbioses: Aphids and their symbiotic bacteria *Buchnera*. Annu. Rev. Entomol..

[11] Moran N.A., Russell J.A., Koga R., Fukatsu T. (2005). Evolutionary relationships of three new species of *Enterobacteriaceae* living as symbionts of aphids and other insects. Appl. Environ. Microbiol..

[12] Zhou X., Ling X., Guo H., Zhu-Salzman K., Ge F., Sun Y. (2021). *Serratia symbiotica* enhances fatty acid metabolism of pea aphid to promote host development. Int. J. Mol. Sci..

[13] Kang Z.W., Zhang M., Cao H.H., Guo S.S., Liu F.H., Liu T.X. (2022). Facultative endosymbiont *Serratia symbiotica* inhibits the apterization of pea aphid to enhance its spread. Microbiol. Spectr..

[14] Nichols H.L., Goldstein E.B., Saleh Ziabari O., Parker B.J. (2021). Intraspecific variation in immune gene expression and heritable symbiont density. PLoS Pathog..

[15] Monnin D., Jackson R., Kiers E.T., Bunker M., Ellers J., Henry L.M. (2020). Parallel evolution in the integration of a co-obligate aphid symbiosis. Curr. Biol..

[16] Perreau J., Patel D.J., Anderson H., Maeda G.P., Elston K.M., Barrick J.E., Moran N.A. (2021). Vertical Transmission at the Pathogen-Symbiont Interface: *Serratia symbiotica* and Aphids. mBio.

[17] Pons I., Scieur N., Dhondt L., Renard M.E., Renoz F., Hance T. (2022). Pervasiveness of the symbiont *Serratia symbiotica* in the aphid natural environment: Distribution, diversity and evolution at a multitrophic level. FEMS Microbiol. Ecol..

[18] Burke G.R., Moran N.A. (2011). Massive genomic decay in *Serratia symbiotica*, a recently evolved symbiont of aphids. Genome Biol. Evol..

[19] Koga R., Tsuchida T., Fukatsu T. (2003). Changing partners in an obligate symbiosis: A facultative endosymbiont can compensate for loss of the essential endosymbiont *Buchnera* in an aphid. Proc. Biol. Sci..

[20] Wang Z.-W., Zhao J., Li G.Y., Hu D., Wang Z.G., Ye C., Wang J.J. (2024). The endosymbiont *Serratia symbiotica* improves aphid fitness by disrupting the predation strategy of ladybeetle larvae. Insect Sci..

[21] Lamelas A., Gosalbes M.J., Manzano-Marin A., Pereto J., Moya A., Latorre A. (2011). *Serratia symbiotica* from the aphid *Cinara cedri*: A missing link from facultative to obligate insect endosymbiont. PLoS Genet..

[22] Montllor C.B., Maxmen A., Purcell A.H. (2002). Facultative bacterial endosymbionts benefit pea aphids *Acyrthosiphon pisum* under heat stress. Ecol. Entomol..

[23] Wang Q., Yuan E., Ling X., Zhu-Salzman K., Guo H., Ge F., Sun Y. (2020). An aphid facultative symbiont suppresses plant defence by manipulating aphid gene expression in salivary glands. Plant Cell Environ..

[24] Emelianoff V., Chapuis E., Le Brun N., Chiral M., Moulia C., Ferdy J.B. (2008). A survival-reproduction trade-off in entomopathogenic nematodes mediated by their bacterial symbionts. Evolution.

[25] Schwenke R.A., Lazzaro B.P., Wolfner M.F. (2016). Reproduction-immunity trade-offs in Insects. Annu. Rev. Entomol..

[26] Renoz F. (2024). The nutritional dimension of facultative bacterial symbiosis in aphids: Current status and methodological considerations for future research. Curr. Res. Insect Sci..

[27] Yao Z., Cai Z., Ma Q., Bai S., Wang Y., Zhang P., Guo Q., Gu J., Lemaitre B., Zhang H. (2022). Compartmentalized PGRP expression along the dipteran *Bactrocera dorsalis* gut forms a zone of protection for symbiotic bacteria. Cell Rep..

[28] Maire J., Vincent-Monegat C., Masson F., Zaidman-Remy A., Heddi A. (2018). An IMD-like pathway mediates both endosymbiont control and host immunity in the cereal weevil *Sitophilus* spp.. Microbiome.

[29] Login F.H., Balmand S., Vallier A., Vincent-Monegat C., Vigneron A., Weiss-Gayet M., Rochat D., Heddi A. (2011). Antimicrobial peptides keep insect endosymbionts under control. Science.

[30] Renoz F., Lopes M.R., Gaget K., Duport G., Eloy M.C., de Merxem B.G., Hance T., Calevro F. (2022). Compartmentalized into Bacteriocytes but Highly Invasive: The Puzzling Case of the Co-Obligate Symbiont *Serratia symbiotica* in the Aphid *Periphyllus lyropictus*. Microbiol. Spectr..

[31] Pang X., Xiao X., Liu Y., Zhang R., Liu J., Liu Q., Wang P., Cheng G. (2016). Mosquito C-type lectins maintain gut microbiome homeostasis. Nat. Microbiol..

[32] Wang W., Wang G., Zhuo X., Liu Y., Tang L., Liu X., Wang J. (2020). C-type lectin-mediated microbial homeostasis is critical for Helicoverpa armigera larval growth and development. PLoS Pathog..

[33] Shigenobu S., Stern D.L. (2013). Aphids evolved novel secreted proteins for symbiosis with bacterial endosymbiont. Proc. R. Soc. B-Biol. Sci..

[34] Uchi N., Fukudome M., Nozaki N., Suzuki M., Osuki K.-I., Shigenobu S., Uchiumi T. (2019). Antimicrobial activities of cysteine-rich peptides specific to bacteriocytes of the pea aphid *Acyrthosiphon pisum*. Microbes Environ..

[35] Loth K., Parisot N., Paquet F., Terrasson H., Sivignon C., Rahioui I., Ribeiro Lopes M., Gaget K., Duport G., Delmas A.F. (2022). Aphid BCR4 structure and activity uncover a new defensin peptide superfamily. Int. J. Mol. Sci..

[36] Nakabachi A., Shigenobu S., Sakazume N., Shiraki T., Hayashizaki Y., Carninci P., Ishikawa H., Kudo T., Fukatsu T. (2005). Transcriptome analysis of the aphid bacteriocyte, the symbiotic host cell that harbors an endocellular mutualistic bacterium, *Buchnera*. Proc. Natl. Acad. Sci. USA.

