# Supplementary material for: Transcriptional Reshaping of Bacteriocytes in the Aphid–Serratia Symbiosis
Source: Insects. 2026 Aug 5;17(8):815. doi: 10.3390/insects17080815 (PMC13512998; doi:10.3390/insects17080815)
Supplement: Supplementary file 1 [file insects-17-00815-s001.zip › Table S1.pdf]

Table S1. Primers used in this study.

	Primers	Sequence 5′→3′
**For diagnostic PCR**		
Buchnera	*Buch16S1*-F	GAGCTTGCTCTCTTTGTCGGCAA
	Buch16S1-R	CTTCTGCGGGTAACGTCACGAA
Serratia	*16S10*-F	AGTTTGATCATGGCTCAGATTG
	16S443-R	CTTCTGCGAGTAACGTCAATG
**For qPCR**		
Buchnera	*BuchdnaK*-F	ATGGGTAAAATTATTGGTATTG
	BuchdnaK-R	ATAGCTTGACGTTTAGCAGG
Serratia	*SradnaK*-F	TGGCGGGTGATGTGAAG
	SradnaK-R	CGGGATAGTGGTGTTTTTGG
Acyrthosiphon pisum	*EF1α*-F	CTGTGCTTATTGTCGCTGCT
	EF1α-R	TCGCTGTATGGTGGTTCAGT
**For FISH**		
Buchnera		Cy5-CCTCTTTTGGGTAGATCC
Serratia		Cy3-CCCGACTTTATCGCTGGC
**For RT-qPCR**		
Rpl37	*qRpl37*-F	CGTAGGAAGACCACCGGAAC
	qRpl37-R	GTTCTGGGCTTAGCTTGGGT
Rpl39	*qRpl39*-F	AGAATCGTCCCATCCCCCA
	qRpl39-R	ACGCCTCTTGGCGTTATATCT
OX	*qOX* -F	CGGGACCACGTAAAGCAGAA

	qOX -R	GCGAGATGGCCTCCTCAAAA
Ndufb2	*qNdufb2*-F	CAAGGCGGTAGCTCCAAGAG
	qNdufb2-R	TCCCATTGAAACGTCGGCAA
Bgm	*qBgm* -F	TTATTTCGGCTGCTGCTCCT
	qBgm -R	ATGTGCACCTCCACACTCA
Fas	*qFas*-F	AAAAACTGTTCGACGGCACG
	qFas-R	ATCTTGGTTACTGCACCGCA
GNBP1	*qGNBP1*-F	GACCAGCAAAGTCGGCAAAA
	qGNBP1-R	CCCATCTTCCATCCTCCACC
GNBP2	*qGNBP2*-F	TGGAAGCCAATCTCCAAACGA
	qGNBP2-R	TCCAGCCACCACCACAAAAT
58977	*q58977*-F	CCGAAGGGAGGGAAAGATGC
	q58977-R	CCACGAGATACTACGCCCAT
64343	*q64343*-F	TCAAGGACGGTGTGTGTCTG
	q64343-R	ATGCGCAACGGGATTGGA
