# Supplementary material for: Transcriptional Reshaping of Bacteriocytes in the Aphid–Serratia Symbiosis
Source: Insects. 2026 Aug 5;17(8):815. doi: 10.3390/insects17080815 (PMC13512998; doi:10.3390/insects17080815)
Supplement: Supplementary file 1 [file insects-17-00815-s001.zip › Table S2.pdf]

Table S2. Data output quality and mapping rates for the examined samples.

Samples	Clean reads	Q30 (%)	GC (%)	Genome Mapping Ratio (%)
Serratia-_1	23,963,687	96.98	39.03	76.38
Serratia-_2	22,564,376	97.20	41.27	76.07
Serratia-_3	25,697,012	96.81	39.60	82.13
Serratia+_1	38,394,084	97.11	40.73	82.65
Serratia+_2	29,269,770	97.20	41.98	90.68
Serratia+_3	41,869,334	97.29	40.08	91.51
